# Supplementary material for: Feasibility of the 30 s Sit-to-Stand Test in the Telehealth Setting and Its Relationship to Persistent Symptoms in Non-Hospitalized Patients with Long COVID
Source: Diagnostics (Basel). 2022 Dec 21;13(1):24. doi: 10.3390/diagnostics13010024 (PMC9818883; doi:10.3390/diagnostics13010024)

Supplementary Materials

Figure S1. Association between exertional fatigue and dyspnea after 30-STS and validated dyspnea and fatigue questionnaires (FAS and LCADL).

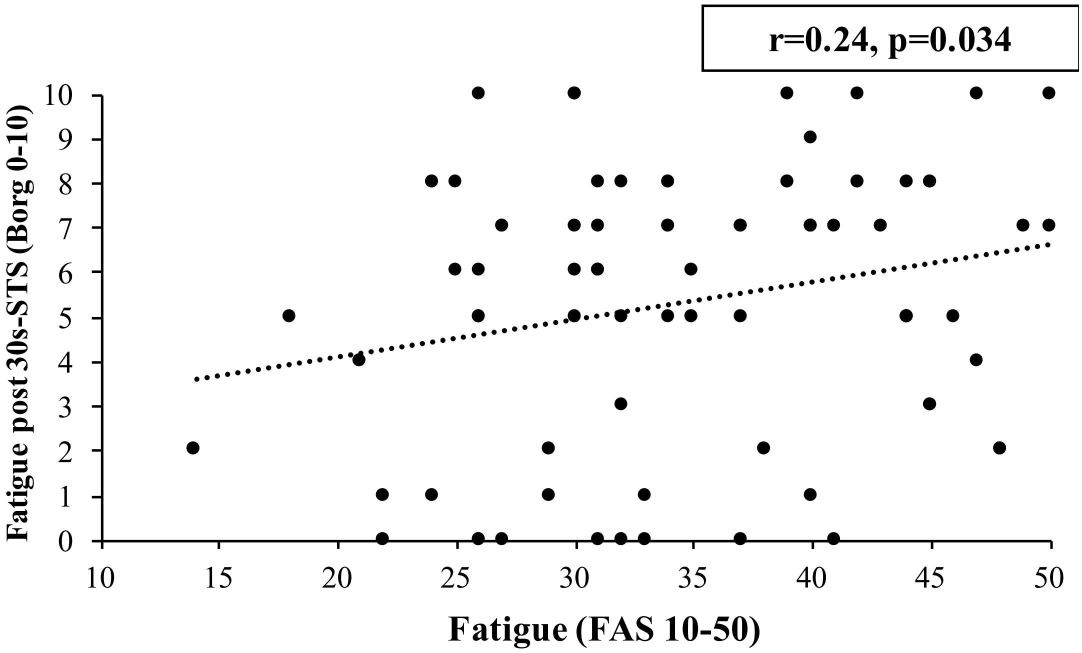

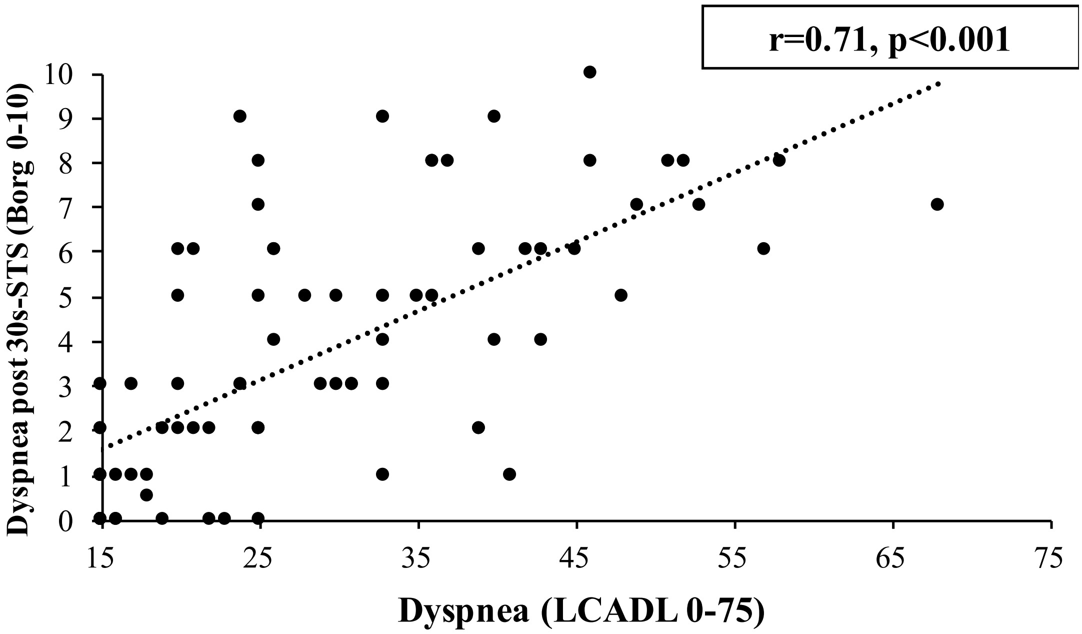

Supplement: Supplementary file 1 [file diagnostics-13-00024-s001.zip › diagnostics-2048489-supplementary.pdf]
